# Gut distress and intervention *via* communications of SARS-CoV-2 with mucosal exposome

**DOI:** 10.3389/fpubh.2023.1098774

**Published:** 2023-04-17

**Authors:** Yuseok Moon

**Affiliations:** ^1^Laboratory of Mucosal Exposome and Biomodulation, Department of Integrative Biomedical Sciences, Pusan National University, Yangsan-si, Republic of Korea; ^2^Biomedical Research Institute, Pusan National University, Busan, Republic of Korea; ^3^Graduate Program of Genomic Data Sciences, Pusan National University, Yangsan-si, Republic of Korea

**Keywords:** COVID-19, SARS-CoV-2, meta-analysis, gastrointestinal symptoms, gut-lung axis, microbiota, nutritional intervention

## Abstract

Acute coronavirus disease 2019 (COVID-19) has been associated with prevalent gastrointestinal distress, characterized by fecal shedding of severe acute respiratory syndrome coronavirus 2 (SARS-CoV-2) RNA or persistent antigen presence in the gut. Using a meta-analysis, the present review addressed gastrointestinal symptoms, such as nausea, vomiting, abdominal pain, and diarrhea. Despite limited data on the gut–lung axis, viral transmission to the gut and its influence on gut mucosa and microbial community were found to be associated by means of various biochemical mechanisms. Notably, the prolonged presence of viral antigens and disrupted mucosal immunity may increase gut microbial and inflammatory risks, leading to acute pathological outcomes or post-acute COVID-19 symptoms. Patients with COVID-19 exhibit lower bacterial diversity and a higher relative abundance of opportunistic pathogens in their gut microbiota than healthy controls. Considering the dysbiotic changes during infection, remodeling or supplementation with beneficial microbial communities may counteract adverse outcomes in the gut and other organs in patients with COVID-19. Moreover, nutritional status, such as vitamin D deficiency, has been associated with disease severity in patients with COVID-19 *via* the regulation of the gut microbial community and host immunity. The nutritional and microbiological interventions improve the gut exposome including the host immunity, gut microbiota, and nutritional status, contributing to defense against acute or post-acute COVID-19 in the gut–lung axis.

## 1. Introduction

The coronavirus disease-19 (COVID-19) first occurred in 2019 and is now a worldwide pandemic with more than 15 million deaths (1). Typically, the presence of gastrointestinal signs or symptoms during COVID-19 has been associated with approximately 35–50% of COVID-19 cases. In a meta-analysis examining 4,243 patients, the pooled prevalence of gastrointestinal symptoms was 17.6% (2). Frequently observed gastrointestinal symptoms include anorexia, diarrhea, vomiting, and abdominal pain (3). With increasing COVID-19 severity, gastrointestinal symptoms were more apparent (4). The pathogenesis of COVID-19, including gastrointestinal symptoms, remains elusive, despite tissue-specific immunofluorescence detection of SARS-CoV-2 binding to a specific receptor such as angiotensin-converting enzyme 2 (ACE2), predominantly expressed in the gastrointestinal tract (5, 6). Numerous cohort studies have reported that patients with COVID-19 and gastrointestinal symptoms might exhibit an increased risk for worse clinical outcomes (7, 8). Disruption of the intestinal mucosal immune barrier can result in gut commensal microbes and pathogens entering local inner tissues and the vascular system, leading to septicemia and acute respiratory distress syndrome (ARDS) (9). Immune cells induced by various antigens can move between the gut and lungs *via* the lymphatic system or blood vessels, thereby regulating the immune response of target organs. Moreover, humoral factors, including cytokines and hormones, contribute to inter-organ communication (10).

The “gut–lung axis” is defined as the cross-talk between intestinal and pulmonary tissues mediated by microbes, immune cells, immune mediators, and other endogenous humoral regulators (11). SARS-CoV-2-induced distress in the gut–lung axis can be elucidated by several potent mechanisms: 1. Viruses directly cause gastrointestinal distress, resulting in symptoms, such as diarrhea, abdominal pain, and vomiting. 2. Viral infection may excessively trigger tissue injury factors, including proinflammatory cytokines, during a cytokine storm, increasing the risk of sepsis, ARDS, and multiorgan failure. 3. Viral infection may dysregulate the intestinal microbiota, increasing the risk of immunological disorders in the gut–lung axis and the systemic impact. Considering the gut–lung axis, we compared the gastrointestinal exposure and underlying pathogenesis mechanisms, including gut barrier distress, mucosal immune dysregulation, and disruption of the microbial community in the gut. Accordingly, the present review addressed the potential role of the gut–lung axis in the pathogenesis of COVID-19 and microbiota alteration in the immune response to establish effective dietary interventions. Inter-organ communication could provide new insights into gut-based interventions against SARS-CoV-2 infection.

## 2. Clinical symptom-based association between viral infection and gastrointestinal adverse outcomes

**Figure 1:**
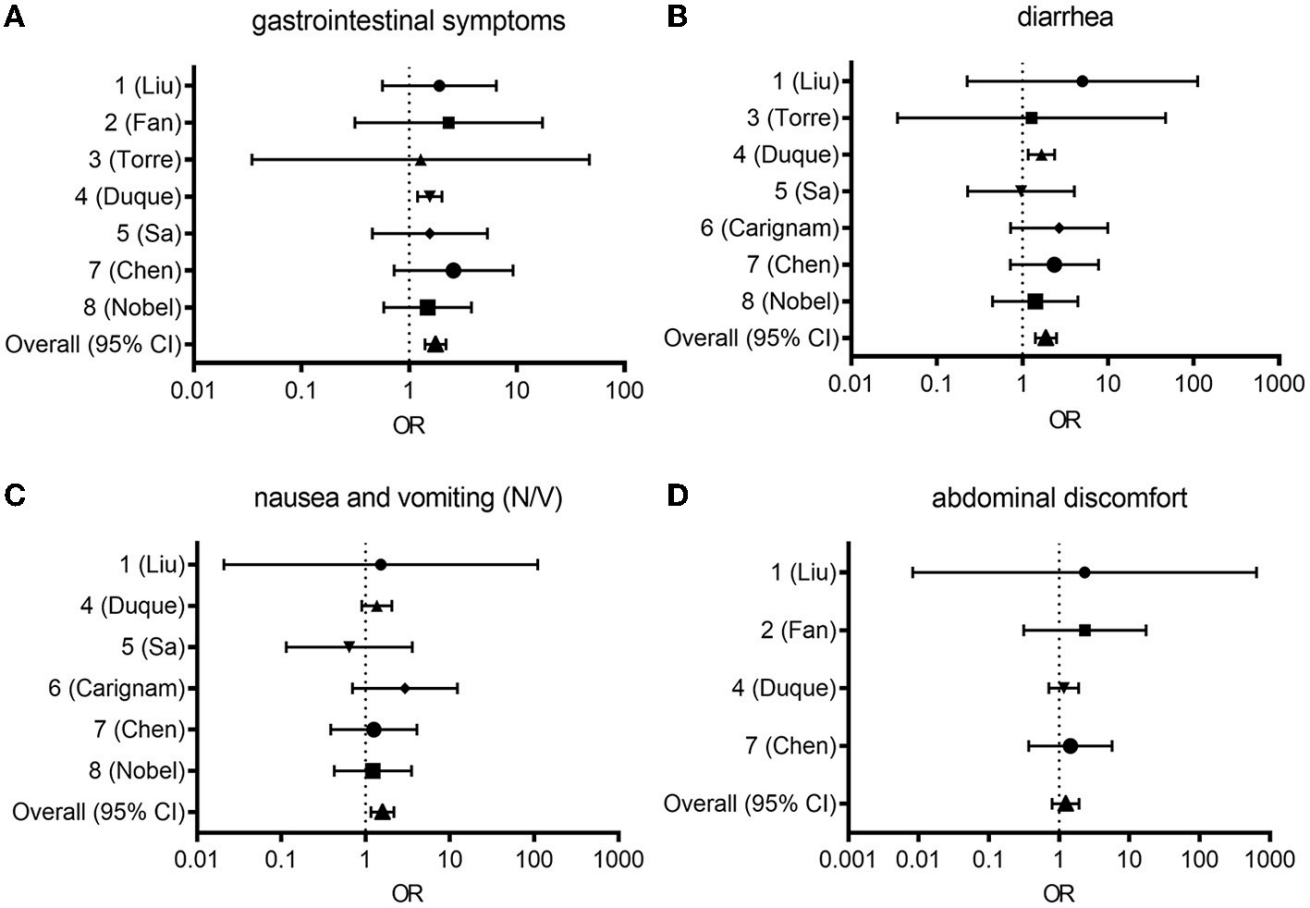
Forest plot from random effects analysis: OR for presenting any gastrointestinal symptom **(A)**, diarrhea **(B)**, nausea and vomiting [N/V, **(C)**], abdominal discomfort **(D)** in the COVID-19 group vs. the control group. CI, confidence interval; COVID-19, coronavirus disease 2019; OR, odds ratio.

First, we evaluated the clinical evidence using the literature-based symptoms of gut distress in patients with COVID-19. The literature search for this association was performed according to the Preferred Reporting Items for Systematic Reviews and Meta-Analyses (PRISMA) guideline. To address the clinical association between SARS-CoV-2 infection and gut distress, we performed the meta-analysis by collecting studies reporting the gastrointestinal symptoms or clinician-observed features in patients using laboratory-confirmed methods. To obtain an evidence-based minimum set of items according to the PRSIMA guideline, the gastrointestinal symptom-based case-control studies were selected from PubMed and LitCovid (*n* = 244), ScienceDirect (*n* = 759), and Google (*n* = 140). After de-duplication, all unique citations were independently screened by reviewers. In particular, articles that failed to meet established inclusion criteria were excluded by screening titles and abstracts, scrutinizing, and the consensus decision-making. We included studies with adequately available data on both control and case groups, but excluding case reports and studies of patients with symptoms other than gastrointestinal symptoms or underlying diseases such as cancer, autoimmune disease, and metabolic diseases. Finally, eight articles were evaluated in the meta-analysis (Figure 1). The selected articles covered events in 14,188 patients, comprising 2,800 COVID-19-positive patients and 11,388 control patients from five countries, including the USA, Portugal, China, Italy, and Australia. For efficient data extraction, we combined symptoms of “abdominal pain” and “abdominal distension” into the more prevalent and widely reported symptoms of “abdominal discomfort”. Where studies reported one symptom “or” another (e.g., nausea or vomiting), we extracted the prevalence of both. We extracted grouped symptoms (e.g., any gastrointestinal symptoms) without further description or definition, rather than using the sum of all gastrointestinal symptom data to prevent data overlapping between symptoms. The pooled prevalence of each symptom was estimated using the Metaprop package and the variance was normalized using a random-effects model such as Freeman-Tukey arcsine transformation of the prevalence. Statistical heterogeneity was assessed by I2, the proportion of total variation due to inter-study heterogeneity.

### 2.1. Association of gastrointestinal symptoms with COVID-19

In study ID 1, the pooled odds ratio (OR) was 1.91 (95% confidence interval [Cl]: 1.17–3.12), with a weight of 15.18% (12). In study ID 2, the pooled OR was 2.34 (95% Cl: 1.94–5.23), with a weight of 13.64% (13). In study ID 3, the pooled OR was 1.28 (95% Cl: 0.30–5.48), with a weight of 9.30% (14). In study ID 4, the pooled OR was 1.56 (95% Cl: 1.40–1.73) with a weight of 16.10% (15). In study ID 5, the pooled OR was 1.5 (95% Cl: 0.95–2.56), with a weight of 15.14% (16). In study ID 7, the pooled OR was 2.59 (95% Cl: 1.55–4.32), with a weight of 15.08% (17). In study ID 8, the pooled OR was 1.49 (95% Cl: 1.02–2.17), with a weight of 15.56% (18). Collectively, the pooled OR of 1.76 (95% CI: 1.61–1.93) indicated a significant association between COVID-19 and GI symptoms, while the random-effect meta-analysis revealed a large heterogeneity among studies (I^2^ = 98.1%; Figure 1A).

### 2.2. Association of diarrhea with COVID-19

In study ID 1, the pooled OR was 5.03 (95% Cl: 1.44–17.53), with a weight of 11.16% (12). Study ID 2 was not included (13). In study ID 3, the pooled OR was 1.28 (95% Cl: 0.30–5.48), with a weight of 9.65% (14). In study ID 4, the pooled OR was 1.67 (95% Cl: 1.45–1.93) with a weight of 16.66% (15). In study ID 5, the pooled OR was 0.96 (95% Cl: 0.54–1.72), with a weight of 15.33% (16). In study ID 6, the pooled OR was 2.69 (95% Cl: 1.59–4.56), with a weight of 15.58% (19). In study ID 7, the pooled OR was 2.37 (95% Cl: 1.47–3.82), with a weight of 15.78% (17). In study ID 8, the pooled OR was 1.42 (95% Cl: 0.89–2.24), with a weight of 15.85% (18). Overall, the pooled OR of 1.88 (95% CI: 1.68–2.11) indicated a significant association between COVID-19 and diarrhea, while the random-effect meta-analysis revealed a large heterogeneity among studies (I^2^ = 96.2%; Figure 1B).

### 2.3. Association of nausea and vomiting with COVID-19

In study ID 1, the pooled OR was 1.53 (95% Cl: 0.27–8.50), with a weight of 9.02% (12). Study IDs 2 and 3 were not included in this analysis (13, 14). In study ID 4, the pooled OR was 1.36 (95% Cl: 1.16–1.61), with a weight of 19.27% (15). In study ID 5, the pooled OR was 0.64 (95% Cl: 0.32–1.28), with a weight of 17.09% (16). In study ID 6, the pooled OR was 2.93 (95% Cl: 1.65–5.23), with a weight of 17.80% (19). In study ID 7, the pooled OR was 1.26 (95% Cl: 0.78–2.01), with a weight of 18.31% (17). In study ID 8, the pooled OR was 1.22 (95% Cl: 0.80–1.87), with a weight of 18.51% (18). Overall, the pooled OR of 1.59 (95% CI: 1.40–1.87) indicated a significant association between COVID-19 and diarrhea, while the random-effect meta-analysis revealed a large heterogeneity among studies (I^2^ = 97.9%; Figure 1C).

### 2.4. Association of abdominal discomfort with COVID-19

In study ID 1, the pooled OR was 2.30 (95% Cl: 0.24–22.38), with a weight of 8.60% (12). In study ID 2, the pooled OR was 2.34 (95% Cl: 1.04–5.23) with a weight of 27.98% (13). Study ID 3 was not included (14). In study ID 4, the pooled OR was 1.17 (95% Cl: 0.96–1.42) with a weight of 32.80% (15). Study IDs 5 and 6 were not included in this analysis (16, 19). In study ID 7, the pooled OR was 1.24 (95% Cl: 0.84–2.52), with a weight of 30.62% (17). Study ID 8 was absent (18). Overall, the pooled OR of 1.24 (95% CI: 1.04–1.48) indicated a significant association between COVID-19 and abdominal discomfort, while the random-effect meta-analysis revealed a large heterogeneity among studies (I^2^ = 96.0%; Figure 1D).

Owing to the high levels of heterogeneity (96.0–98.1%) among studies, additional subgroup analysis, meta-regression, or sensitivity analysis could clarify the underlying causes behind high heterogeneity between studies. The Newcastle-Ottawa Scale may afford an alternate tool for assessing the quality of case-control studies in meta-analyses (20). Taken all symptoms and prevalence, all pooled OR (95% CI: 1.04-2.24) indicated notable positive associations between COVID-19 and gut distress-associated symptoms despite the heterogeneity between studies. Based on the literature-based assessment of the clinical outcomes, we further evaluated the pathological processes and mechanisms of the lung-gut communications in patients with COVID-19.

## 3. Viral entry and translocation into the gut–lung axis

### 3.1. Airway entry and reverse translocation to gut

Coronaviruses are enveloped single-stranded RNA viruses characterized by club-like spikes projecting from their surfaces, with a remarkably large RNA genome. The SARS-CoV-2 genome encodes four major structural proteins: spike (S), nucleocapsid (N), membrane (M), and envelope (E), each of which is essential for composing the viral particle (21). Phylogenetic analysis of the complete genome sequence of SARS-CoV-2 revealed that the new virus shares 89.1% nucleotide sequence identity with SARS-like coronaviruses detected in bats (22). ACE2, the functional receptor of SARS-CoV-1 and SARS-CoV-2, plays a crucial role in the pathogenesis of COVID-19, as it allows viral entry into human cells (23). Similar to SARS-CoV-1, the viral S protein of SARS-CoV-2 binds to ACE2 as a cellular receptor. Importantly, SARS-CoV-2 is more pathogenic, partly owing to its 10-to-20-fold increased binding affinity for ACE2 (24). This binding leads to viral host cell entry, in parallel with S protein priming by the host cell protease, transmembrane serine protease 2 (TMPRSS2). The S glycoprotein contains two functional domains: an S1 receptor-binding domain (RBD) and a second S2 domain that mediates the fusion of viral and host cell membranes (25). The SARS-CoV-2 S protein initially binds to the ACE2 receptor on the host cell through the S1 RBD. The S1 domain is shed from the viral surface, allowing the S2 domain to fuse with the host cell membrane. This process depends on the activation of the S protein by cleavage at two sites (S1/S2 and S2') *via* the proteases furin and TMPRSS2. Furin-induced cleavage leads to conformational changes in the viral S protein, exposing the RBD and S2 domains. TMPRSS2-mediated cleavage of the SARS-CoV-2 S protein facilitates the fusion of the viral capsid with the host cell to permit viral entry (26). Exposure of the RBD in the S1 protein subunit results in an unstable subunit conformation; thus, during binding, this subunit undergoes conformational rearrangement between two states, known as the up- and down-conformations. The down-state transiently hides the RBD, whereas the up-state exposes the RBD but temporarily destabilizes the protein subunit (27–29). Within the trimeric S protein, only one of the three RBD is present in an accessible conformation for binding with the ACE2 receptor.

ACE2 is detected in the nasal and bronchial epithelial cells. In addition to the upper respiratory tract, ACE2 is abundantly expressed on the surface of alveolar type II pneumocytes, which co-express several other genes involved in the regulation of viral reproduction and transmission, including TMPRSS2. Type II pneumocytes are well-known to produce surfactants, maintain their self-renewal ability, and exert immunoregulatory functions. Importantly, these cells share the same basement membrane as the closely juxtaposed capillary endothelial cells, which also express high ACE2 levels. Therefore, type II pneumocytes, along with the neighboring capillary endothelium, could be primary sites for SARS-CoV-2 entry, resulting in damage to the alveolocapillary membrane with reactive hyperplasia of type II pneumocytes. As type II pneumocytes are known targets of viral entry and replication, this may lead to a vicious cycle of persistent alveolar wall destruction and repair, eventually culminating in progressive, severe diffuse alveolar damage. Upregulated ACE2 expression has been documented in the airways of patients with chronic respiratory disease who are smokers, which, together with disturbed ciliary movement and abnormal mucus viscosity, may increase disease vulnerability (30). However, clinical evidence indicates that smoking does not necessarily lead to increased vulnerability (31). Recently, a healthy human donor-based evaluation suggested that the virus could exploit goblet and ciliated cells in the nasal epithelia as entry portals, a plausible primary infection site (32). Considering the variant-mediated adverse outcomes, Omicron is known to cause relatively mild symptoms compared with other variants of concern. The Omicron variant can enter epithelial cells through different binding proteins such as cathepsins and display lower replication competence than other variants (33), potently contributing to attenuated severity of the clinical outcomes.

Airway particles, including viral particles, are entrapped in the airway mucosa and cleared *via* mucociliary transport. However, the clearance system can be damaged following SARS-CoV-2 infection *via* dedifferentiation of multiciliated cells and subsequent attenuation of cilial movement, as shown in a reconstructed human bronchial epithelium model (34). As guardians of the airway, alveolar macrophages can play crucial roles in removal *via* phagocytosis or translocation from the peripheral lung to the larynx, with subsequent passage through the gut and fecal excretion (35). In addition to gastrointestinal translocation from the airway, the virus can enter the water and food supply systems directly, ultimately reaching the gastrointestinal tract in humans (36, 37). Viral particles that successfully reach the alveolar vasculature or translocate into the gut can systematically affect extra-airway tissues including the gut if they escape the immune system in circulation.

### 3.2. Vascular translocation and circulation of SARS-CoV-2

ACE2 receptors are also expressed in endothelial cells. It remains unknown whether vascular derangements in COVID-19 can be attributed to endothelial cell involvement mediated by the virus. Intriguingly, SARS-CoV-2 can directly infect engineered human blood vessel organoids *in vitro* (38). In this *in vitro* experiment, to verify the possibility of COVID-19 transmission through the endothelial tissue, the authors used human capillary organoids from induced pluripotent stem cells infected with SARS-CoV-2 (39). Notably, human recombinant secretory ACE2 could inhibit infection in organoids mimicking human capillaries with CD31 and PDGFR.

An initial study has suggested that the SARS-CoV-2 S protein can bind to CD147 on the cell surface and subsequently enter blood cells, such as platelets and megakaryocytes. Megakaryocytes and platelets actively take up SARS-CoV-2 virions, possibly through an ACE-2-independent mechanism. Based on *in vitro* antiviral tests, meplazumab, an anti-CD147 humanized antibody that blocks the interaction between the S protein and the CD147 cell surface receptor, could significantly inhibit viral cell entry into circulation. CD147 is a SARS-CoV-2 surface entry receptor, leading to inflammation and thrombosis, which differs from the common cold coronavirus. Moreover, given that elevated blood sugar levels could upregulate CD147 expression, diabetes could be a potential risk factor for poor prognosis in patients with COVID-19 (40). Vasculature-translocated surviving viral particles are available for the secondary tissue infection and subsequent inflammatory outcomes in the gut.

### 3.3. Gut entry *via* fecal–oral transmission

Owing to intestinal viral RNA shedding, there have been growing concerns that SARS-CoV-2 could be transmitted *via* the fecal–oral route, given that viral RNA has been detected in patient stool samples (41). It has been suggested that the presence of gastrointestinal symptoms is a likely indicator of viral RNA in the stool (2, 42). In contrast, studies have failed to establish a statistically significant correlation between viral RNA and increased gastrointestinal symptom intensity (41, 43). However, it has been suggested that stool samples may be positive for viral RNA even when the virus is undetectable in respiratory samples (2, 44). It is well-established that viruses can enter the gut, but most cannot survive in the digestive tract, owing to the low pH of gastric fluid and the harsh intestinal environment comprising bile and digestive enzymes. Therefore, no infectious virus was recovered from the fecal samples of patients with COVID-19. Although stool is unlikely to contain infectious viruses (45), confirmative assessments are warranted to comprehensively establish the risk of fecal–oral transmission during infection and its significance in the food system (46).

Theoretically, SARS-CoV-2 directly invades the gastrointestinal epithelium through ACE2 receptor. ACE2 is highly expressed in the esophageal upper and stratified epithelium, as well as in absorptive enterocytes derived from both the ileum and colon (5). In approximately 50% of COVID-19 cases, viral RNA was detected in fecal samples, even in the absence of gastrointestinal tract manifestations and after clearance of respiratory infection, thereby suggesting an asymptomatic SARS-CoV-2 infection in the gut and the possibility of fecal–oral transmission (47). However, considering the limited data available, a fecal–oral transmission route clarifying enteric symptoms in patients with COVID-19 is yet to be proposed. Moreover, it is also challenging to rationalize that SARS-CoV-2, as an enteric virus, passes through the stomach and reaches the intestine to infect the intestinal cells. For successful infection *via* fecal–oral transmission, the virus must overcome biological barriers, such as stomach acid and intestinal bile salts after ingestion. Coronavirus can undergo complete inactivation at pH 2.26 and 4.38 at 37 °C (48). Although the virus can survive under wet or dry conditions for up to 3 days, it was found to survive at pH 2.2 for up to 1 h only at high concentrations (49). Bile salts are one of the various mechanisms that mediate host defense, exerting detergent action against the lipid layer integrity of infectious agents (50). SARS-CoV-2 contains an outer lipid-containing membrane and is an enveloped virus (23). Bile acid is known to be effective against viruses with lipoproteins, but envelope-deficient variants are resistant to its detergent action. Considering all the evidence, in addition to the airway viral infection, the oral ingestion of surviving viral particles contributes to the gastrointestinal distress.

## 4. Impact of SARS-CoV-2 on mucosal defense

### 4.1. SARS-CoV-2-mediated gut barrier distress

The gut is divided into several anatomical barriers, each of which plays a vital role in serving as a barrier against foreign materials, such as pathogens and other noxious stimuli. The mucus layer is the first line of defense, composed of mucus, antibodies, and other antimicrobial factors (51). It functions as a physical barrier protecting epithelial cells from microbes (bacteria, fungi, and virus) and large molecules, such as food particles (52). The second layer, beneath the mucus layer, comprises highly glycosylated proteins, glycocalyx, lining the epithelial cell surface. These cell membrane-bound glycoproteins, such as the mucus layer, act as a physical barrier that prevents pathogenic microorganisms from communicating with the gut epithelial cellular monolayer and invading the submucosal tissues (53). The epithelial cell barrier is another defense mechanism against gut microbes and luminal antigens *via* modulation of the epithelial junctional molecules or transmitting danger signals to the underlying mucosal immune system while facilitating the transport of nutrients and water (54). Epithelial cells have pattern recognition receptors (PRRs), such as Toll-like receptors (TLRs), which allow the recognition of microbial antigens. Enterocytes (or intestinal epithelial cells) are the most common cell type in the mucosal epithelial layer, accounting for 90% of cells (55). Enterocytes are well-known absorption sites and important components of the gut barrier. Gut epithelial cells can also interact with SARS-CoV-2 through the highly expressed ACE2 (24, 56, 57). SARS-CoV-2 has been shown to infect intestinal organoids (58). Furthermore, TMPRSS2, which is also highly expressed in enterocytes in the ileum and colon (57, 59), reportedly participates in priming the SARS-CoV-2 S protein and facilitates viral entry into cells (24). Accordingly, ACE2 and TMPRSS2 are promising targets for intervention against SARS-CoV-2 (60) despite limited evidence on the efficacy of blockers targeting the two proteins (61). Intestinal viral infections may damage the epithelial barrier. For example, Middle East respiratory syndrome-related coronavirus was shown to disrupt the gut epithelial barrier in an animal model (62). Mechanistically, SARS-CoV infection can lead to the redistribution of the PALS1 protein, a tight junction protein, and subsequent disruption of epithelial integrity in the gut and lungs. Moreover, SARS-CoV-2 RNA and viral nucleocapsid protein were persistent in mucosal tissues and cells, including the gut epithelium and CD8^+^ T cells of patients with inflammatory bowel disease nearly 7 months after SARS-CoV-2 infection (63). Consistent with the airway infection, the Omicron variants showed reduced levels of cytotoxicity- and damage-associated markers in infected gut organoids, compared with the wild type virus and delta variants (33). In contrast, delta variant-infected mini-gut exhibited active clustering of infected gut cells and relatively high levels of the replication efficacy. Since active invasion by the Omicron variant was extremely scarce and lumen-restricted in the gut model, the variant is not assumed to affect the submucosa parts. Therefore, different strains may have different relative tissue tropisms and invasiveness, potently leading to strain-specific clearance rates and clinical symptoms in the gut.

Fecal SARS-CoV-2 RNA has been detected in 50% of patients experiencing gastrointestinal symptoms, such as abdominal pain, nausea, and vomiting, within the first week after diagnosis (64). In particular, 12.7% (8.5–18.4%) of subjects displayed persistent fecal shedding of SARS-CoV-2 RNA even after 4 months of diagnosis, without ongoing shedding of oropharyngeal SARS-CoV-2 RNA. Although the above-mentioned study failed to link mucosal viral antigens with the severity of acute COVID-19, it is necessary to address the roles of mucosa-persistent antigens in mucosal defense, recurrence, and disease progression as post-acute sequelae of COVID-19 (PASC). After acute COVID-19, most patients with inflammatory bowel disease presented persistent presence of SARS-CoV-2 antigens in their gut mucosa, irrespective of inflammation levels, potentially contributing to PASC symptoms (63). Despite the lack of mechanistic evidence, it has been proposed that SARS-CoV-2 may increase intestinal permeability, potentially by damaging enterocytes and the epithelial layer (65), necessitating further molecular investigation.

### 4.2. Mucosal and systemic innate immunity to SARS-CoV-2

Coronaviruses are known to cause airway damage and lead to pneumonia with imbalanced and hyper-immune responses (22). Increased proinflammatory cytokines and lymphocytopenia have been associated with severe acute respiratory syndrome coronavirus 2 (SARS-CoV-2) infection (66). An unbalanced immune response and excessive inflammatory cytokine secretion, known as a “cytokine storm,” have been associated with disease severity and worse prognosis in patients with COVID-19, including multiorgan failure (67, 68). Of 197 patients, approximately 34.5% presented neutrophilia (69), which is known to trigger ARDS and sepsis growth in patients with COVID-19. Secondary hemophagocytic lymphohistiocytosis (SHLH), an underrecognized hyperinflammatory syndrome, could also be a significant factor in the development of COVID-19, given that SHLH can cause hypercytokinemia-related fatal and fulminant multiorgan failure (70).

SARS-CoV-2 can spread *via* respiratory droplets, contact, and the fecal–oral route. Viral replication commences in the nasopharynx and upper respiratory tract and continues through the lower respiratory tract and gastrointestinal mucosa (5). Monocytes, macrophages, and dendritic cells (DCs) can serve as primary hallmarks of SARS-CoV-2 infection, given that they link innate and adaptive immunity and play an important role in the antiviral response (71–73). Although the precise correlation between DCs and SARS-CoV-2 in the mucosa has been poorly explored, SARS-CoV-2 accelerates the activation of PRR-linked signaling, including NLRP3 inflammasome or occasionally leads to the cytokine release syndrome (CRS) *via* robust production of proinflammatory mediators, such as interleukin (IL)-6, granulocyte-macrophage colony-stimulating factor, IL-1β, and tumor necrosis factor (TNF)-α during the CRS (74). Therapeutic agents, such as anti-IL-6R, which can target macrophage-related activity, could be crucial interventions against the cytokine storm that occurs during severe SARS-CoV-2 infection (33). In addition to the phagocytic system, natural killer (NK) cells have been associated with a severely poor prognosis of SARS-CoV-2 infection in the presence of functional exhaustion. Among the various cytokines produced during early severe COVID-19, interferon (IFN)-α expression markedly correlated with the severity of COVID-19 (75, 76). According to single-cell transcriptomic analysis based on two COVID-19 cohorts, IFN-α directly suppressed IFN-γ production by NK cells (76). Moreover, exhausted NK cells reportedly express CD94/NK group 2 member A(NKG2A), which functions as an inhibitory receptor that reduces the production of CD107a, IFN-γ, IL-2, granzyme B, and TNF-α. Therefore, improving NK cell-mediated defense might be a promising defense mechanism during early severe cases of SARS-CoV-2 infection (77, 78). Active NK cells recognize viral infection and transmit death signals into the infected cells in the mucosa. Moreover, NK cells may facilitate mucosal phagocyte-induced viral clearance *via* production of anti-virus cytokines including type I interferons. However, exhausted NK cells would fail to defend against SARS-CoV-2 in the mucosa.

### 4.3. Acquired immunity and mucosal vaccination against SARS-CoV-2

In addition to direct infective actions of SARS-CoV-2, respiratory virus-responsive mucosal and systemic acquired immune responses would affect the disease progression in the extra-airway tissues. Cytotoxic CD8+ T cells directly neutralize infected cells or CD4+ T cells initiate a humoral response by cooperating with B cells (79, 80). During severe SARS-CoV-2 infection, lymphopenia is accompanied by a marked reduction in CD4+ T and CD8+ T cells, along with elevated neutrophil counts (81–83). An increased neutrophil-to-lymphocyte ratio and elevated levels of IL-6 can indicate poor prognosis and disease severity. Increased serum levels of proinflammatory cytokines, such as IL-6, IL-7, IL-1β, IL-2, and IL-10, can induce a cytokine storm and cause serious damage, more destructive than the coronavirus itself. Elevated proinflammatory cytokine levels have been linked to viral sepsis, respiratory failure, shock, and even death if severe (84). Therefore, addressing lymphopenia and cytokine storm could prevent severe complications associated with coronavirus.

Following the appearance of COVID-19 symptoms, the antibody response increases after 4–8 days, and IgM becomes predominant (85), followed by 10–18 days of persistent IgA and IgG production. IgA is crucial in mucosal defense by neutralizing SARS-CoV-2 and weakening the inflammatory risk (86). The antigen can attach to intestinal epithelial cells or microfold (M) cells, followed by transport into lymph nodes and IgA-secreting B cell activation in the lymphoid tissue (87, 88). Considering SARS-CoV-2, the antigen amount and quality critically impact neutralization. Antibodies should be specific to the S protein and must be detected in the serum for 2–3 weeks post-infection (89, 90). Human convalescent serum transfer has been proposed as a potential strategy to prevent and treat severe cases of COVID-19, with its therapeutic value documented in several clinical trials (84, 91–94). An important challenge in overcoming COVID-19 is viral elimination from the mucosa through antibody-associated shedding. Given that infectious agents trigger mucosal immunity (95), mucosal vaccination could be a promising strategy to evoke IgA antibodies at both the mucosal surface and the systemic immune system (96). Importantly, mucosal vaccination may facilitate IgA-virus complex formation in the mucosa of respiratory and intestinal tissues (97). As current modes of COVID-19 vaccination are predominantly based on systemic antigen exposure, efficient strategies are needed to develop promising mucosal vaccination against continuously evolving SARS-CoV-2.

## 5. Involvement of gut microbial community in SARS-CoV-2 pathogenesis

Following initial lung infection, SARS-CoV-2 invades the gut mucosal immune barrier, directly impacting the intestinal physiology. Moreover, intestinal tissue damage may facilitate gut dysbiosis. It has been reported that commensal microbiota in the lung and gut can counterbalance viral infection by modulating immune responses in a homeostatic manner (98, 99). For instance, viral infection-induced changes in pulmonary tissues and other microenvironments may alter the structure and function of the gut microbiota (98). In a mouse model, seasonal influenza infection of the respiratory tract increased the number of *Enterobacteria* in the gut microbiota and decreased the number of *Lactobacillus* and *Lactococcus* (99). Furthermore, intestinal dysbiosis has been associated with increased mortality following respiratory infections, probably due to deregulated airway immune responses. Inflammatory dysbiosis of the gut microbiota and epithelial damage reportedly enhance ACE2 levels, increasing the risk of SARS-CoV-2 infection in the gastrointestinal tract, as well as dissemination to other sites *via* circulation (5, 100).

### 5.1. Microbiota-linked prediction of adverse outcomes

Various studies have revealed how SARS-CoV-2 infection can alter gut microbiota and its association with adverse outcomes in humans. In particular, viral infection-altered gut communities were shown to be associated with inflammatory status in patients with COVID-19. Serum-based proinflammatory biomarkers positively correlated with increased levels of some consortia, including *Ruminococcus gnavus*, during viral infection, whereas *Clostridia* was negatively correlated (101). Moreover, disease severity could be correlated with the abundance of *Coprobacillus, Clostridium ramosum*, and *Clostridium hathewayi* (102). It has been reported that approximately 50% of patients with COVID-19 display stool positivity for SARS-CoV-2 even in the absence of gastrointestinal manifestations and after recovery of respiratory SARS-CoV-2 infection (47), indicating the presence of persistent gut infection. Based on viral infectivity prediction using metagenomic analysis of the fecal SARS-CoV-2 genome, patients with COVID-19 demonstrate an increased functional capacity for nucleotide and amino acid biosynthesis and carbohydrate metabolism (47). An in-depth assessment demonstrated an evident correlation between viral infection signatures and the enrichment of gut pathogens, including *Collinsella aerofaciens, Collinsella tanakaei, Streptococcus infantis*, and *Morganella morganii*, even in the absence of gastrointestinal manifestations (47). Although the Omicron variant is known to cause relatively mild symptoms with marginal invasiveness in humans and gut models, all SARS-CoV-2 variants of concerns remarkably disrupted the mouse gut microbiota (103). Surprisingly, the Omicron variant infection led to long-lasting instability in the gut microbiota and a notable depletion in *Akkermansia muciniphila*, even in the absence of severe lung pathology. In addition to host markers or disease severity, the fecal viral footprint was notably associated with dysbiosis-linked alterations in gut bacterial communities, paving the way for novel diagnostic tools for potent relapse or chronic adverse outcomes in post-COVID or long-term COVID conditions, potently with differential responses to SARS-CoV-2 variants.

In addition, SARS-CoV-2 infection can alter the gut virome community. Although patients with COVID-19 presenting reduced abundance exhibit an under-representation of RNA virus and multiple bacteriophage lineages (DNA viruses), they have notable gut enrichment of environment-derived eukaryotic DNA viruses, mainly including crAs-like phages, *Myoviridae*, and *Siphoviridae* families, even after of 30 days of symptom resolution (104, 105). Viral genes involved in bacteriophage integration, DNA repair, metabolism, and virulence are predicted to contribute to host stress and inflammation; however, some viral consortia are inversely associated with blood levels of proinflammatory proteins, white cells, neutrophils, and disease severity (104, 105). These resident enteric viruses maintain a low level of immune stimulation and are responsible for protective and regulatory effects in the intestine (106). However, given the limited data on the effects of viral composition on microbiota composition and activity during SARS-CoV-2 infection, advanced interkingdom associations need to be addressed to improve the integrated prognosis and intervention against adverse outcomes in patients with post-COVID or long COVID.

### 5.2. Microbiota-based probiotic counteraction against infection

In patients with COVID-19, reduced beneficial commensals were directly correlated with disease severity and complications (107). It is speculated that a decline in probiotic intestinal microbiota would fail to effectively control excessive proinflammatory immune reactions, leading to the subsequent progression of SARS-CoV-2 infection. Considering the immunomodulatory cytokine production in response to beneficial commensal bacteria, the abundance of *Lactobacillus* species decreased in correlation with anti-inflammatory IL-10 levels during SARS-CoV-2 infection (108). Therefore, serum IL-10 can be employed as a diagnostic indicator to assess disease progression and severity in high-risk patients with COVID-19 (108). Moreover, disease severity is inversely correlated with the abundance of *Faecalibacterium parusnitzii*, an anti-inflammatory bacterium (102) and subjects with low levels of viral infectivity features presented a relatively high abundance of short-chain fatty acid-producing beneficial bacterial communities, including *Parabacteroides, Bacteroides, Alistipes*, and *Lachnospiraceae*, even in the absence of gastrointestinal manifestations (47). Furthermore, several gut immune-modulating commensal bacteria, including *Faecalibacterium prausnitzii, Eubacterium rectale*, and *bifidobacteria*, were inversely associated with levels of proinflammatory mediators, tissue injury markers (lactate dehydrogenase, aspartate aminotransferase, and gamma-glutamyl transferase), and disease severity (109). Accordingly, these immune-modulating bacteria can potentially counteract proinflammatory and toxic insults during viral infection, providing novel insights into interventions against adverse outcomes during PASC conditions. Patients with PASC tended to display high levels of *Ruminococcus gnavus and Bacteroides vulgatus* and low levels of *Bifidobacterium pseudocatenulatum* and *Faecalibacterium prausnitzii* (110). Considering the inflammatory states due to reduced levels of probiotic commensal community, patients with COVID-19 are speculated to be remarkably susceptible to infection by opportunistic bacteria, such as *Klebsiella pneumoniae, Streptococcus*, and *Ruminococcus gnavus*, particularly during the hospitalization period (102). Likewise, patients with PASC were found to be markedly susceptible to nosocomial gut pathogens, such as *Clostridium innocuum* and *Actinomyces naeslundii* (110). These opportunistic bacteria can potentially trigger the production of proinflammatory cytokines, such as IFN-γ and TNF-α (102). Overall, the reduced abundance of probiotic gut bacteria can be associated with severe inflammatory responses *via* the excessive production of proinflammatory cytokines and severe complications in high-risk patients with COVID-19. Therefore, remodeling or supplementation with beneficial microbial communities are promising interventions against the gut mucosal distress in patients with COVID-19.

## 6. Effects of nutritional status on susceptibility to COVID-19

### 6.1. Association of nutritional deficiency with disease severity during viral infection

Considering the gastrointestinal involvement in SARS-CoV-2 infection, dietary components, including nutrients, bioactive natural products, and probiotics, were assumed to contribute to immune regulation in response to viral infections. In the French NutriNet-Santé cohort study assessing 7,766 adult patients with anti-SARS-CoV-2 antibodies, dietary intake of vitamin C, vitamin B9, vitamin K, fibers, and fruit vegetables was associated with lower susceptibility to SARS-CoV-2 infection, whereas dietary intake of calcium and dairy products did not contribute to the infection risk (111). The beneficial effects of vitamin C have been well-documented in various *in vitro* and *in vivo* studies. Exposure to high doses of vitamin C can induce antiviral actions against various viruses (112). In clinical trials, treatment with a high dose of intravenous (IV) vitamin C decreased vasopressor requirements and improved mortality in patients with septic shock (113). In addition to intervention against non-communicable chronic diseases *via* regulation of inflammation and complications, various dietary components, including vitamin C treatment, can contribute to the supportive clinical management of infectious diseases, such as COVID-19 (114).

**Figure 2:**
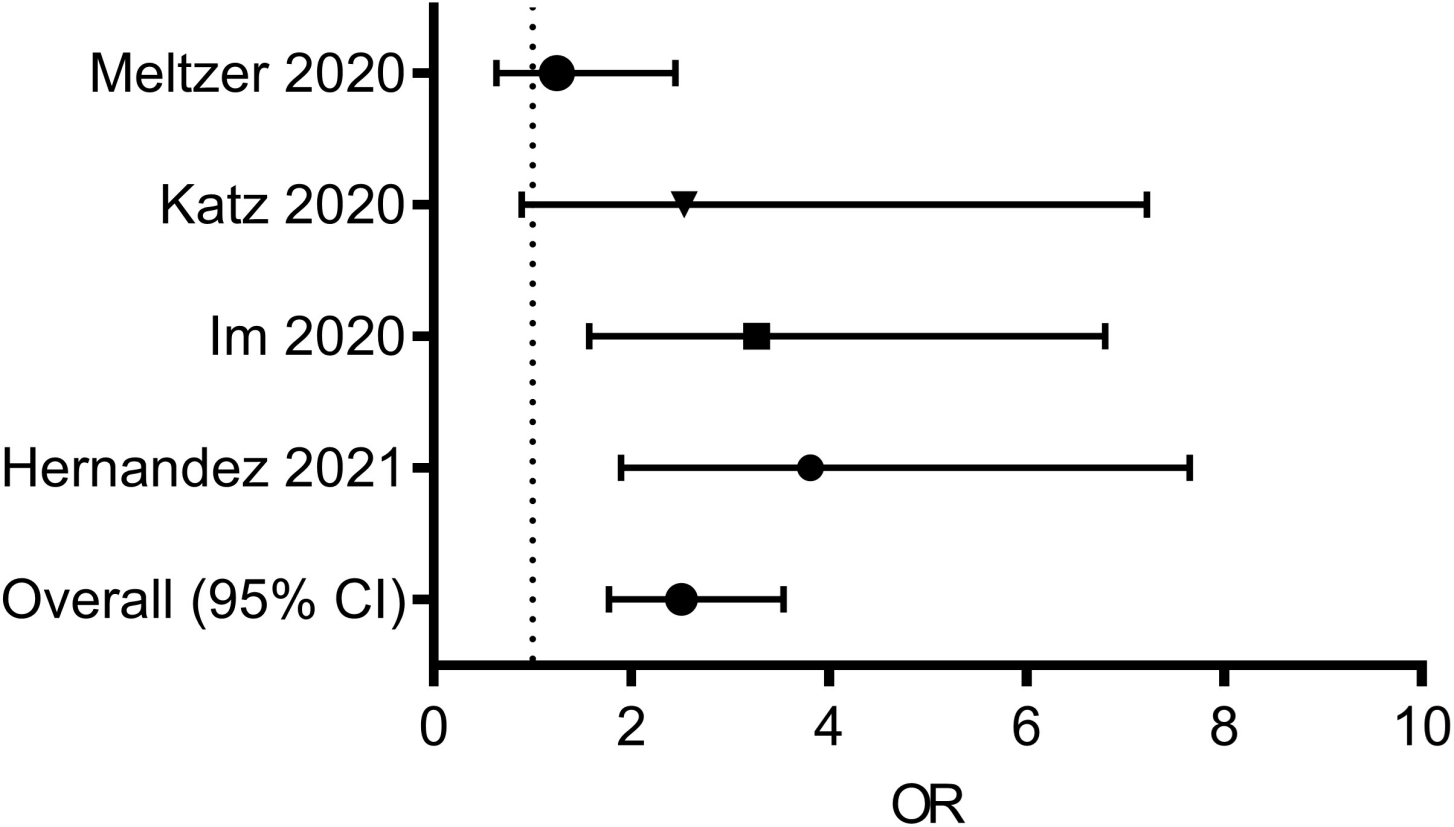
Forest plot from random effects analysis: Vitamin D status (Low serum 25OHD, daily dietary intake) and COVID-19 infection rate. ORs of having vitamin D deficiency in the COVID-19 group vs. the control group. 25OHD, 25-hydroxyvitamin D; COVID-19, coronavirus disease 2019; ORs, odds ratio.

In addition to vitamin C, multiple lines of evidence suggest a potential link between vitamin D and SARS-CoV-2 infection (115–118). Vitamin D is an essential lipid-soluble nutrient absorbed from dietary sources in the proximal small intestine, contributing to skeletal management, intestinal calcium absorption, and immune regulation (119). Although vitamin D deficiency was associated with respiratory distress in patients hospitalized for pneumonia (120), the association between low vitamin D intake and disease severity in COVID-19 cases remains poorly explored (121). A retrospective cohort study revealed that vitamin D deficiency status was positively associated with an increased COVID-19 risk (115). Another retrospective case-control study assessed the possible influence of vitamin D status on disease severity in hospitalized patients with COVID-19 (116). Serum 25-hydroxyvitamin D (25OHD) levels were lower in hospitalized patients with COVID-19 than those in population-based controls, and these patients presented a higher prevalence of vitamin D deficiency (116). Severe vitamin D deficiency (based on a cut-off of ≤ 10 ng/dL) was noted in 24.0% of patients in the COVID-19 group when compared with 7.3% in the control group (117). Another study by the University of Florida revealed that patients with vitamin D deficiency were five times more likely to be infected with COVID-19 than those without deficiency after adjusting for age groups (118). Taken together, dietary status, such as vitamin D deficiency, may present a risk factor for COVID-19 susceptibility and severity (Figure 2). Moreover, the association of the amount, duration, and interval of nutrient intake with disease severity and prevalence needs to be examined. In addition, specific pathophysiological mechanisms of dietary factor-linked protection should be examined to clarify adverse outcomes in patients.

### 6.2. Nutritional intervention against gut defense deterioration during viral infection

Vitamin D may counteract gut distress by improving the mucosal and epithelial barriers. Vitamin D supplementation and activation of its nuclear receptor (vitamin D receptor [VDR]) can improve epithelial barrier integrity by enhancing the expression of VDR-associated intracellular junction proteins, including occludin, claudin, and zonula occludens, in the distressed gut (122, 123). Conversely, vitamin D deficiency may compromise the mucosal barrier (124), leading to an increased susceptibility to mucosal damage and infection risk in patients with COVID-19. Moreover, the synthesis and secretion of antimicrobial peptides were elevated *via* vitamin D metabolite-linked VDR activation or subsequent activation of TLR1/2 signaling in the mucosa (125, 126), thereby regulating the excessive commensal bacteria and pathogens by the epithelium or mucosal immune system. Moreover, vitamin D supplementation can activate non-canonical pathways involving the aryl hydrocarbon receptor (AhR), facilitating epithelial tight junctions and mediating anti-inflammatory and antioxidant actions in the injured gut barrier (127). Collectively, vitamin D and the activation of its nuclear receptors, including VDR or AhR, could improve the gut mucosal and epithelial barrier during SARS-CoV-2 infection.

### 6.3. Nutritional intervention against gut dysbiosis during viral infection

**Figure 3:**
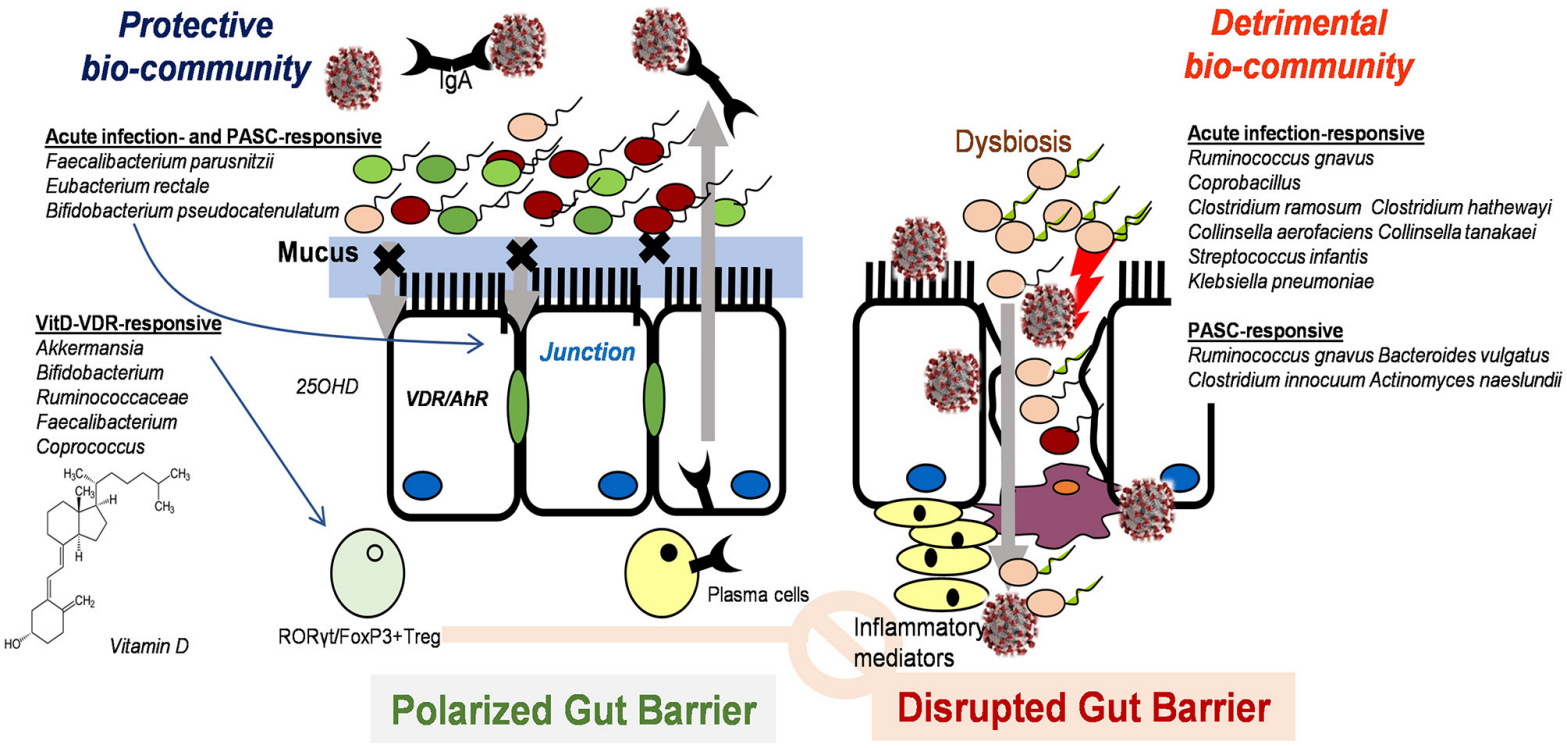
Postulated scheme of vitamin D-induced intervention against SARS-CoV-2 infection in the gut mucosa *via* modulation of microbiota and subsequent immune regulation.

In addition to the direct effects of vitamin D on gut cell physiology, nutritional supplementation is speculated to act on the gut microbial community as another mucosal exposome during SARS-CoV-2 infection. In various experimental models and human studies, notable correlations have been documented between vitamin D and gut microbiota (128, 129). Vitamin D supplementation in healthy individuals significantly increases gut microbial diversity, with an increased ratio of the phylum *Bacteroidetes* to *Firmicutes* (128). Moreover, vitamin D supplementation could remarkably enhance the abundance of health-promoting probiotic taxa, including *Akkermansia, Bifidobacterium, Ruminococcaceae, Faecalibacterium*, and *Coprococcus*, while a significant decrease in *Bacteroides acidifaciens* was observed in non-responders. In particular, some probiotic genera, such as *Lactobacillus reuteri*, can metabolize vitamin D to 7-dehydrocholesterol *via* bile salt hydrolase, subsequently contributing to the pools of circulating 25OHD (130). Moreover, supplementation with 25OHD reportedly attenuates inflammatory responses in experimental models of inflammatory bowel disease, accompanied by gut microbial regulation (131). Mechanistically, compared with vitamin D-deficient subjects, vitamin D-sufficient animals displayed enhanced levels of gut microbe-responsive RORγt/FoxP3+ regulatory T cells in the colon. Notably, the number of anti-inflammatory regulatory T cells positively correlated with the abundance of Bacteroides and Clostridium XIVa. Overall, vitamin D status was predicted to shape the gut microbial community, which can facilitate the bioactive metabolic conversion of vitamin D and regulatory responses against inflammation during SARS-CoV-2 infection (Figure 3).

## 7. Conclusions

Gastrointestinal symptoms are reportedly associated with poor outcomes in patients with acute and post-acute COVID-19. Moreover, persistent remaining viral antigens in the gut mucosal tissue present a risk of recurrent, chronic COVID, and post-acute COVID complications. Based on the findings of a meta-analysis, gastrointestinal symptoms, such as diarrhea, nausea, vomiting, and abdominal discomfort, were notably associated with SARS-CoV-2 infection. In addition to gastrointestinal translocation from the airway in the gut–lung axis, the virus can transmit to water and food supply systems directly and ultimately reaches the gastrointestinal tract in humans *via* fecal–oral transmission. Despite the lack of mechanistic evidence, SARS-CoV-2 could disrupt the mucosal and epithelial barrier and reach the circulation and systemic immune system. Moreover, the prolonged presence of viral antigens and disruption of mucosal immunity may increase gut microbial and inflammatory risks, leading to pathological outcomes and post-acute COVID-19 symptoms. In addition to host immune cell regulation, SARS-CoV-2 infection may alter the gut microbial community, potentially shaping the immunological profile during infection. Generally, patients with COVID-19 exhibit lower bacterial diversity and a higher relative abundance of opportunistic pathogens, such as *Klebsiella pneumoniae, Streptococcus*, and *Ruminococcus gnavus* in their gut microbiota than healthy controls. Despite the dysbiotic changes during infection, enhancing specific bacterial communities, such as *Lactobacillus* and *Faecalibacterium parusnitzii*, may counteract adverse inflammatory outcomes in the gut and other organs. Moreover, nutritional status, such as vitamin D deficiency, has been associated with disease severity in patients with COVID-19 *via* regulation of the gut microbial community and mucosal immunity. Vitamin D is predicted to improve the gut mucosal and epithelial barrier by activating its nuclear receptors during SARS-CoV-2 infection. Moreover, vitamin D status is predicted to shape the gut microbial community, which can facilitate the bioactive metabolic conversion of vitamin D and immune regulatory responses against infection-induced inflammatory storms. Herein, the collated evidence provides systemic insights into nutritional and microbiological interventions against acute or post-acute COVID-19 in the gut–lung axis.

## Author contributions

YM contributed to supervision, conceptualization, methodology, formal analysis, visualization, writing, review, and editing.
